# Revisiting the Pacific Meridional Mode

**DOI:** 10.1038/s41598-018-21537-0

**Published:** 2018-02-16

**Authors:** Malte F. Stuecker

**Affiliations:** 1 Department of Atmospheric Sciences, University of Washington, Seattle Washington, USA; 2 Cooperative Programs for the Advancement of Earth System Science (CPAESS), University Corporation for Atmospheric Research (UCAR), Boulder Colorado, USA

## Abstract

Numerous studies demonstrated that the Pacific Meridional Mode (PMM) can excite Central Pacific (CP) El Niño-Southern Oscillation (ENSO) events and that the PMM is mostly a stochastic phenomenon associated with mid-latitude atmospheric variability and wind-evaporation-SST feedback. Here we show that CP sea surface temperature (SST) variability exhibits high instantaneous correlations both on interannual (ENSO-related) and decadal (Pacific Decadal Oscillation (PDO)-related) timescales with the PMM. By prescribing an idealized interannual equatorial CP ENSO SST forcing in a partially-coupled atmosphere/slab ocean model we are able to generate a realistic instantaneous PMM response consistent with the observed statistical ENSO/PMM relationship. This means that CP ENSO and the PMM can excite each other respectively on interannual timescales, strongly suggesting that a fast positive feedback exists between the two phenomena. Thus, we argue that they cannot be considered two independent dynamical entities. Additionally, we show that the interannual CP ENSO SST forcing generates atmospheric circulation variability that projects strongly on the Aleutian Low and North Pacific SST anomalies that exhibit the characteristic PDO pattern.

## Introduction

The Pacific is home to climate variability on sub-annual to interannual timescales associated with the El Niño-Southern Oscillation (ENSO)^1,2^ and its modulation by the annual cycle^3,4^, as well as on longer decadal-to-multi-decadal timescales^5,6^ associated with the Pacific Decadal Oscillation (PDO)^7,8^, the North Pacific Gyre Oscillation (NPGO)^9,10^, and the Interdecadal Pacific Oscillation (IPO)^11^. Much attention has been paid to these modes of climate variability due to their societal impacts and their potential utilization for seasonal-to-decadal climate predictions.

Recent research demonstrated that ENSO variability exhibits pronounced spatial diversity^12–17^. To characterize this spatial diversity, two types of ENSO events have been classified based on the zonal location of their maximum sea surface temperature (SST) anomalies, a so-called Eastern Pacific (EP) El Niño and a Central Pacific (CP) El Niño. This classification has been justified by different teleconnection patterns and corresponding climate impacts associated with them^17^. It is important to note that common CP ENSO indices exhibit pronounced variance both on interannual and decadal timescales^16,18^.

Theoretical research has shown that the tropical coupled ocean-atmosphere system allows for the coexistence of two distinct dynamical ENSO modes, named after their dominant timescales: Quasi-Quadrennial (QQ) and Quasi-Biennial (QB) ENSO^19^. Based on their spatial patterns, time evolutions, and spectral characteristics, it has been hypothesized that the observed EP and CP ENSO types might be manifestations of the QQ and QB dynamical modes respectively^16,20^.

An important statistical mode of climate variability is the so-called Pacific Meridional Mode (PMM)^21^, a coupled SST-surface wind pattern in the Northeastern Pacific that has been proposed to largely originate from stochastic atmospheric forcing in the mid-latitudes^10,21^. The strong coupling between SST and surface winds in the Northeastern Pacific can be explained by the wind-evaporation-SST (WES) feedback mechanism proposed by Xie & Philander^22^. An analogous meridional mode can also be found in the South Pacific^23^. Closely related to the PMM, it has been proposed that mid-latitude stochastic atmospheric variability can affect tropical ENSO variability via the seasonal footprinting mechanism^24^. Several studies showed that the PMM can excite ENSO variability^10,25^ and that ENSO and the PMM together are able to induce decadal variability in the Pacific^10^. Additionally, Thomas & Vimont^26^ demonstrated that the PMM forcing effect on ENSO is nonlinear, meaning that El Niño events are more easily initiated by PMM forcing than La Niña events. Importantly, they also found that a large spread of the PMM forcing effectiveness on ENSO existed within their model ensemble.

An emerging hypothesis is that much of the observed CP ENSO variability might be explained by stochastic forcing via extra-tropical meridional modes and related atmospheric circulation patterns^27,28^. However, somewhat in contrast to these results, a recent predictability study showed that the PMM adds some skill in predicting EP El Niño events, while it provided less skill in predicting CP El Niño events^29^. Another study pointed to a potentially non-stationary behavior of the ENSO/PMM relationship, arguing that after the 1990s atmospheric variability in the North Pacific became more effective in initiating CP El Niño events^30^. A competing hypothesis is that SST variability in the Atlantic Ocean can be responsible for both interannual and decadal^31–33^ climate variability in the Central Pacific. Furthermore, high-frequency Westerly Wind Events (WWE) have been linked to the existence of CP ENSO^34,35^. Regardless of what is causing CP ENSO variability, it has been shown that CP El Niño events are able to induce extra-tropical atmospheric circulation changes (projecting mostly on the North Pacific Oscillation – NPO) that drive decadal changes of the NPGO^36^.

**Figure 1:**
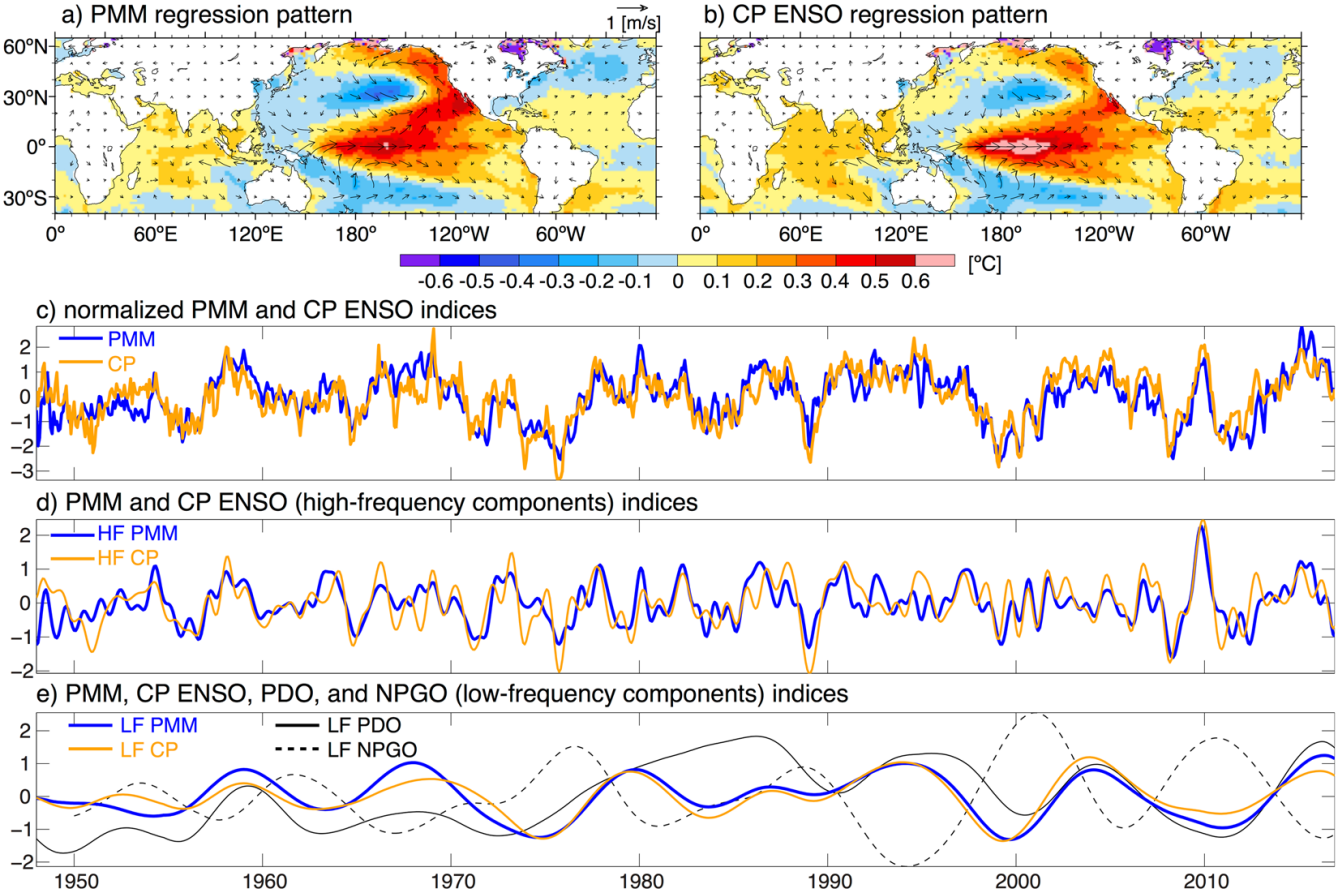
**(a**,**b)** Instantaneous SST (°C) and surface wind (m/s) regression patterns for the anomalous normalized (**a**) PMM and (**b**) CP ENSO indices. **(c)** Time series of the anomalous normalized PMM (blue) and CP ENSO (orange) indices (no units) from 1948–2016. **(d)** Same as (**c**) but for the high-frequency (HF) components of these indices. **(e)** Same as (**c**) but for the low-frequency (LF) components of these indices. Shown additionally are the LF components of the PDO (solid black) and NPGO (dashed black) indices. The maps in this figure were created using NCAR Command Language Version 6.4.0 (10.5065/D6WD3XH5).

If we assume as a hypothesis that CP ENSO events either arise mostly due to local equatorial coupled ocean-atmosphere dynamics in the Pacific or are mostly forced remotely from the Atlantic Ocean, then we have to reconcile this with the observed close statistical relationship between the PMM and CP ENSO events (Fig. 1). Here we set out to provide the missing puzzle piece to explain the causal relationship between the PMM and CP ENSO. First, we are revisiting their observed statistical relationship. Second, we demonstrate with a partially-coupled (PARCP) climate model experiment that the statistical PMM pattern and time evolution can arise in response to equatorial CP ENSO SST forcing without a significant time delay. This indicates a strong coupling and fast positive feedback between the two climate phenomena in nature. Therefore, we argue that CP ENSO and the PMM cannot be considered two independent climate modes. Additionally, in agreement with previous studies^10^ we find that high-latitude Pacific decadal variability can be induced by integrating tropical CP ENSO forcing. The projection of this low-frequency variability on the PMM spatial pattern might explain the observed decadal signal in CP SSTs. Note that throughout this paper we characterize the PMM by using the raw (for which the ENSO signal is not removed) SST expansion coefficient-based PMM index, which differs from the most commonly used PMM index definition that aims to remove some of the ENSO signal (for details refer to the Methods section).

## Observed CP ENSO and PMM Characteristics

The spatial patterns of the SST and surface wind anomalies associated with the PMM (Fig. 1a) and CP ENSO (Fig. 1b) exhibit striking similarities. Note that our PMM regression pattern shows no cold SST anomalies in the eastern equatorial Pacific compared to the original PMM pattern^21^, which is likely due to our choice to use the 1948–2016 anomalies to obtain the pattern instead of the shorter data period used in the original study (1948–2001).

**Figure 2:**
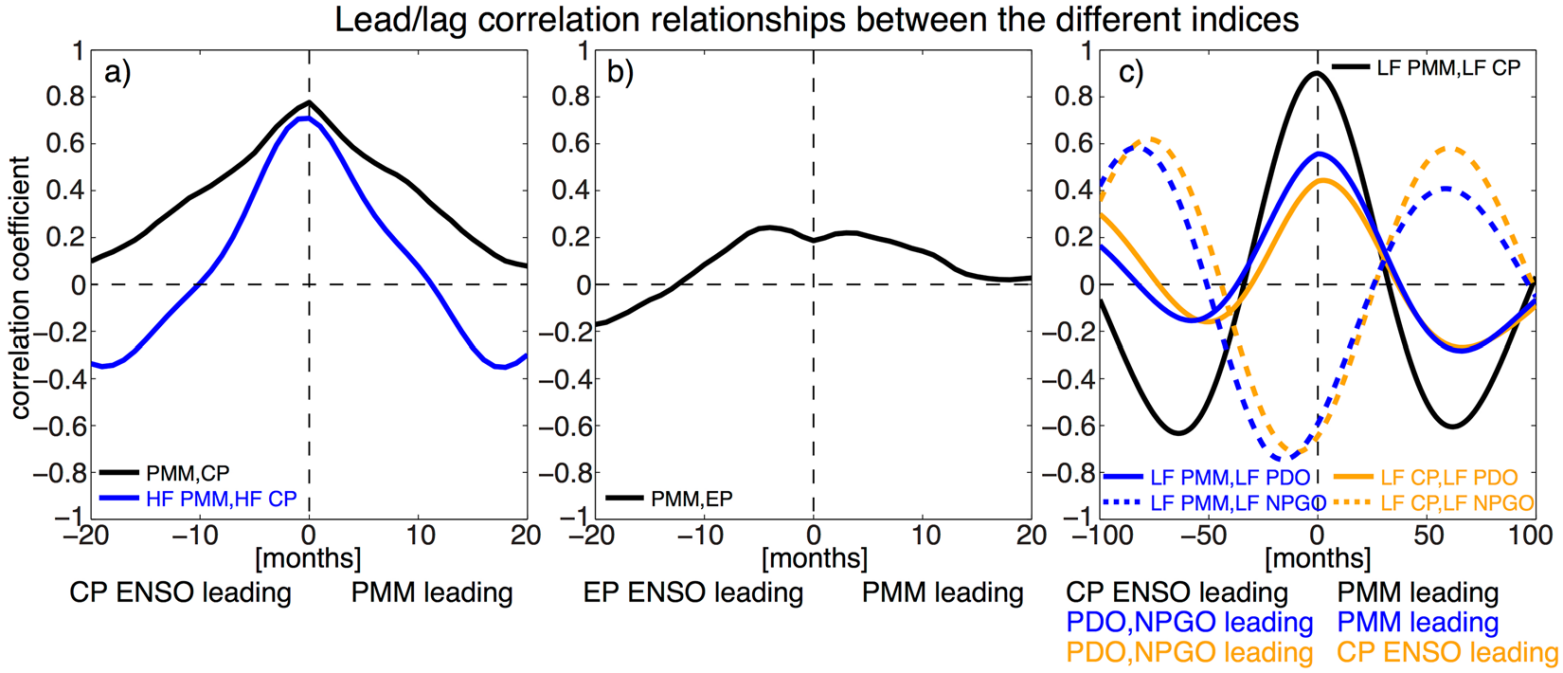
**(a)** Lead/lag correlation between the CP ENSO and PMM indices (solid black) and between the high-frequency components HF CP ENSO and HF PMM (solid blue). **(b)** Lead/lag correlation between the EP ENSO and PMM indices (solid black). **(c)** Lead/lag correlation between the low-frequency components LF CP ENSO and LF PMM (solid black), LF PMM and LF PDO (solid blue), LF PMM and LF NPGO (dashed blue), LF CP ENSO and LF PDO (solid orange), and LF CP ENSO and LF NPGO (dashed orange).

The time evolutions of the PMM and CP ENSO indices exhibit a close agreement with each other (Fig. 1c), with their highest cross-correlation at zero lag (R = 0.78, significant at the 99% confidence level; Fig. 2a). Both PMM and CP ENSO indices can be well separated into high-frequency (HF; Fig. 1d) and low-frequency (LF; Fig. 1e) components via Singular Spectrum Analysis (SSA; see Methods). Again, the highest cross-correlations between these indices exists at zero lag for both timescales (Fig. 2a,c). Furthermore, we observe a relatively close instantaneous relationship between the LF PMM and LF CP ENSO components with the LF PDO and an out-of-phase relationship with the LF NPGO (Figs. 1e and 2c). The close agreement between PMM and CP ENSO also holds on all timescales when looking at the SST and surface wind regression patterns for various lead and lag times (Figs. S1–S3), with no clear evidence that one phenomenon is leading the other.

These results illustrate a few important points about the CP ENSO relationship with the PMM: (i) CP ENSO and the PMM are closely related on both interannual (HF) and decadal (LF) timescales, (ii) no causal statement about which phenomenon causes which can be made from this observational evidence alone, and (iii) the low-frequency components of both indices seem to be related to North Pacific decadal variability associated with the PDO and NPGO. Furthermore, hardly any correlation exists between our EP ENSO index and the PMM, neither instantaneous nor at various lead or lag times (Fig. 2b).

If we want to explain this observed close instantaneous relationship between PMM and CP ENSO on all timescales, we need to reconcile the following results from previous studies: (i) the PMM can effectively induce CP ENSO events^21^, (ii) the occurrence of CP ENSO events might be related to the dynamical QB mode^16,19,20^, (iii) CP SST variability can be induced by remote Atlantic forcing^31–33^, and (iv) CP ENSO events can be induced by stochastic WWE activity^34,35^. These seemingly competing hypotheses can only be reconciled if PMM variability can be effectively induced by equatorial CP ENSO SST forcing. If we can demonstrate this dynamical pathway, then this means that a fast positive feedback mechanism must exist between CP ENSO and the PMM.

## Simulated CP ENSO and PMM Relationship

**Figure 3:**
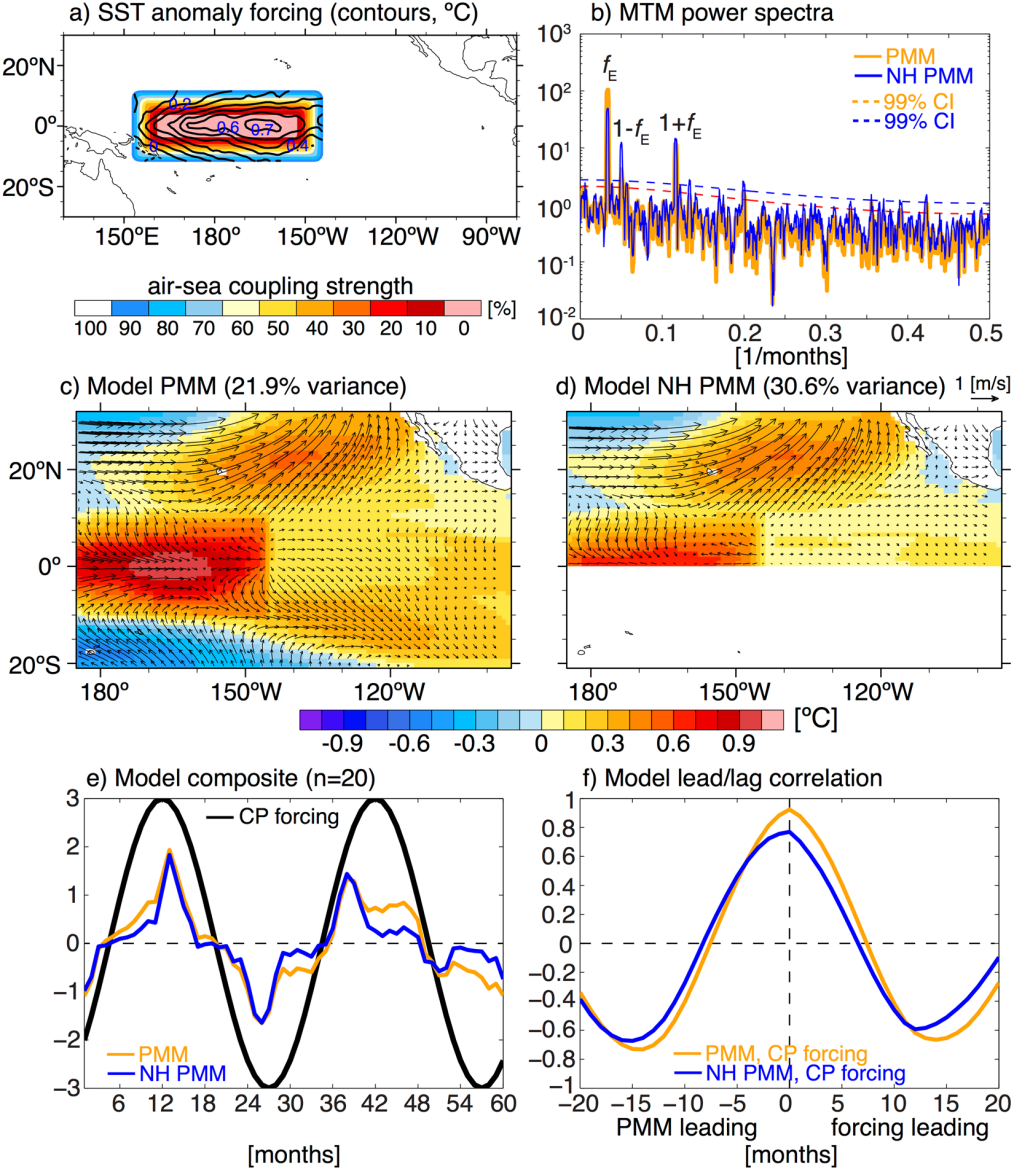
**(a)** CP ENSO SST anomaly forcing pattern (contours, °C) and the PARCP air-sea coupling mask (shading, %). **(b)** MTM power spectra (no unit) for the normalized PMM (solid orange) and NH PMM (solid blue) indices from the full (non-composite) model experiment. Dashed lines indicate the 99% confidence interval for an AR(1) null hypothesis. The forcing frequency (*f*_*E*_) and the first-order combination tones (1 ± *f*_*E*_) are labeled. **(c**,**d)** SST (shading, °C) and low level wind (vectors, m/s) regression patterns for the leading statistical modes of the full model simulation for the traditional PMM domain (**c**) and for the Northern Hemisphere (NH) PMM domain (**d**) respectively. **(e)** Composite time evolution of the CP ENSO forcing (black, no unit) and the normalized simulated PMM (orange, no unit) and NH PMM (blue, no unit) indices. **(f)** Lead/lag correlation between the composite CP ENSO forcing and the composite PMM (orange) and NH PMM (blue) indices respectively. The maps in this figure were created using NCAR Command Language Version 6.4.0 (10.5065/D6WD3XH5).

To test our positive feedback hypothesis we utilize the aforementioned PARCP experiment (see Methods). An idealized CP ENSO SST boundary forcing is only applied in a small region in the Central Pacific (Fig. 3a), while anomalous heat fluxes are able to change the ocean mixed layer temperature (and thereby SST) outside of this forcing region. This PARCP AGCM/slab ocean framework, while being simple, comprises the essential physics (i.e. WES feedback) necessary to generate PMM variability. Extracting the leading statistical mode of coupled anomalous low level wind and SST variability in the region from 175°E–95°W and 21°S–32°N (explaining 21.9% of the total variance) reveals the characteristic PMM spatial pattern, however with loading both in the Northern and Southern Hemispheres (Fig. 3c). The arguably most important feature of the PMM – the weakened trade winds and associated warm SST anomalies in the Northeastern Pacific – is well simulated. To test the robustness of our results we also apply the same statistical decomposition on only the Northern Hemisphere (NH) part of the PMM domain (175°E–95°W and 0°–32°N) and obtain a very similar pattern (explaining 30.6% of the total variance; Fig. 3d).

Next, we composite the corresponding normalized principal components (PC1s) – labelled PMM and NH PMM respectively – according to the prescribed CP ENSO cycle (Fig. 3e). The two simulated PMM indices are very similar to each other but display a slightly different amplitude response mostly in the boreal fall season, indicating nonlinear impacts of CP ENSO on the meridional modes in the Northern and Southern Hemispheres respectively. Importantly, both composite PMM indices are highly correlated with the CP ENSO forcing at zero lag time (Fig. 3f). This clearly demonstrates that meridional modes can arise in both Hemispheres in response to CP ENSO SST forcing by allowing thermodynamic air-sea coupling in the rest of the domain.

Calculating the power spectra for the normalized full (non-composite) PMM and NH PMM indices, we see that most power is located at the 2.5 years CP ENSO forcing periodicity (labeled *f*_*E*_ in Fig. 3b). However, we also observe significant power at ENSO/annual cycle combination tone frequencies (labeled 1 ± *f*_*E*_ in Fig. 3b) arising from a modulation by the annual cycle^3,4^. The higher frequency variability associated with these combination tones can explain the in-seasonal reversals of the PMM indices evident in the composite plot (Fig. 3e). We also observe a slight reddening effect in the spectrum as expected from the integrating effect of the slab ocean. These model results provide support for our initial hypothesis that CP ENSO variability on interannual timescales can induce an instantaneous deterministic PMM response, thereby allowing for a fast positive feedback between CP ENSO and PMM variability, which then would explain their tight coupling that we see in the observations.

## Implications for Decadal Variability

**Figure 4:**
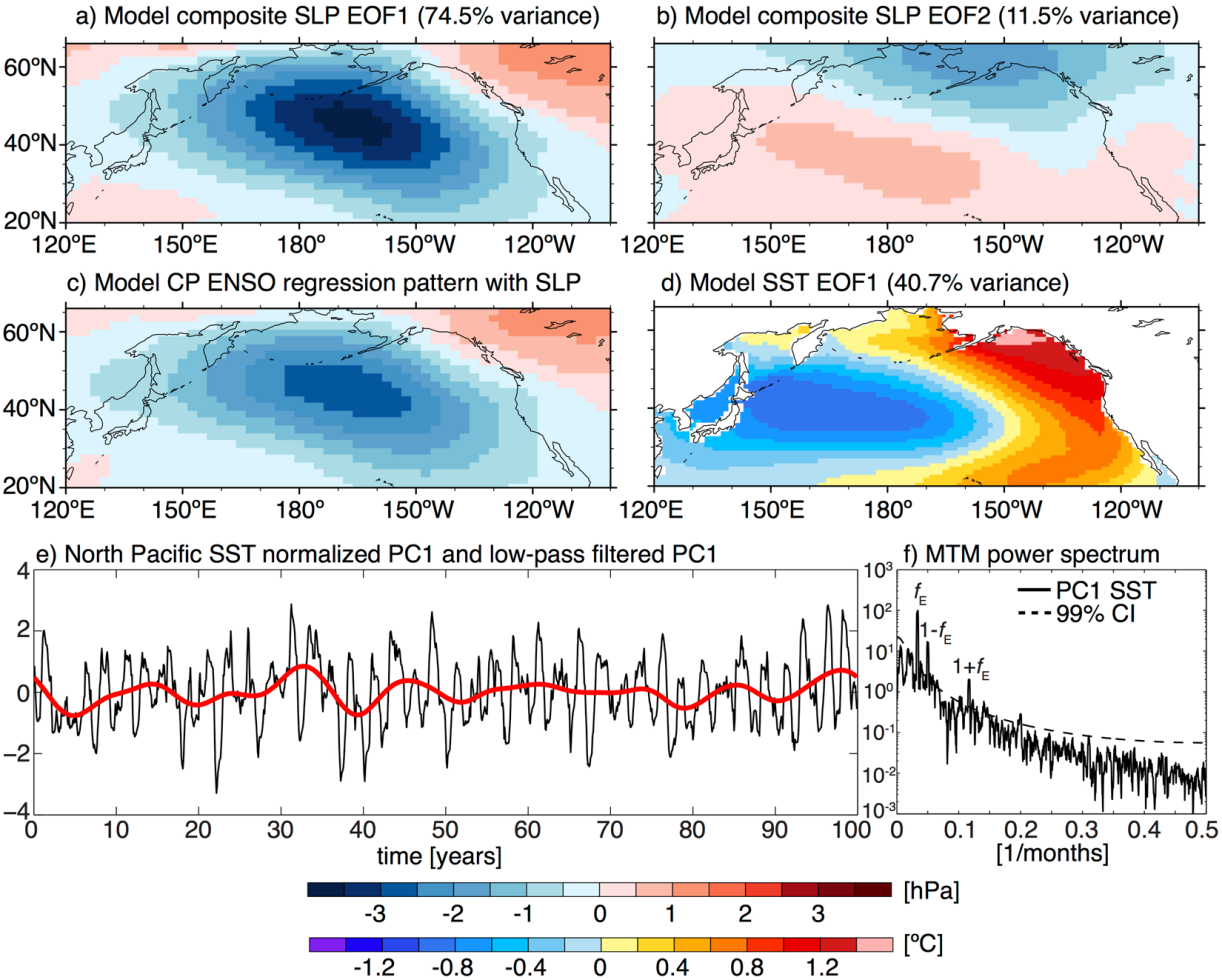
**(a**,**b)** Leading two EOF modes of model composite simulated SLP anomaly variability in the North Pacific (shading, hPa). EOF1 (**a**) projects on the Aleutian Low and EOF2 (**b**) has a structure associated with the North Pacific Oscillation (NPO). **(c)** North Pacific simulated SLP anomaly regression pattern (shading, hPa) for the full (non-composite) model normalized CP ENSO SST anomaly forcing time series. **(d)** Leading North Pacific SST anomaly EOF (shading, °C) for the full model simulation. **(e)** Corresponding normalized PC1 (black) and the 8 years low-pass filtered PC1 (red). **(f)** MTM power spectrum (no unit) for the normalized PC1. The dashed line indicates the 99% confidence interval for an AR(1) null hypothesis. The forcing frequency (*f*_*E*_) and the first-order combination tones (1 ± *f*_*E*_) are labeled. The maps in this figure were created using NCAR Command Language Version 6.4.0 (10.5065/D6WD3XH5).

To investigate the possible influences of the CP ENSO SST forcing on decadal variability we next calculate the leading two Empirical Orthogonal Functions (EOFs) of the model composite simulated North Pacific sea level pressure (SLP) anomalies to focus only on the forced Northern Hemisphere teleconnection patterns. The leading two modes exhibit patterns of Aleutian Low variability (EOF1: explaining 74.5% of the total variance; Fig. 4a) and a meridional pressure seesaw (EOF2: explaining 11.5% of the total variance; Fig. 4b) corresponding to the NPO, respectively (the same leading EOF patterns also appear when the analysis is conducted with the full (non-composite) SLP anomalies, explaining 35.9% and 21.3% of the total variance respectively). The dominant Aleutian Low teleconnection pattern also emerges when calculating the regression coefficient of the periodic CP ENSO SST forcing time series with the full (non-composite) simulated SLP response in the North Pacific (Fig. 4c). It is important to note that the leading pattern obtained here is the Aleutian Low variability (EOF1), while previous studies mostly linked CP ENSO variability to the NPO teleconnection pattern^10,37^. Note also that the relative magnitude of the different teleconnection patterns in the observations depends on which exact climate indices are used in the analysis. Also, mean state and annual cycle biases in different models likely affect the relative importance of these teleconnection patterns in model simulations, which should be further explored with targeted sensitivity experiments in the future.

**Figure 5:**
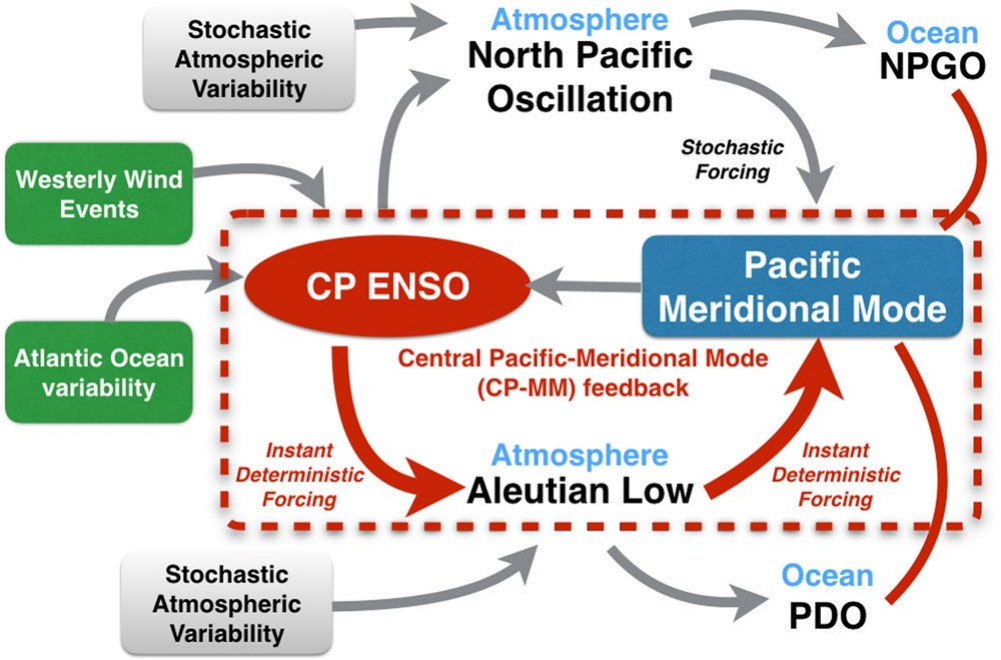
Schematic for the discussed mechanisms (motivated by Fig. 3 in Di Lorenzo *et al*.^10^). Grey arrows indicate previously identified pathways (refer to the Introduction section for details) and red arrows the new pathways proposed in this study that enable an instantaneous positive feedback between CP ENSO and PMM (encircled by the dashed red line), which is able to explain the observed close CP ENSO/PMM relationship at zero lag on all climate timescales. The solid red lines indicate close relationships between PMM, PDO, and NPGO at low-frequencies. Note that stochastic processes can affect (in differing strength) each of the pathways indicated by arrows.

The leading EOF (EOF1) of the model simulated full SST anomalies in the North Pacific exhibits the characteristic pattern associated with the PDO (Fig. 4d). As expected, most variance of the leading PC (PC1; black line in Fig. 4e) is explained by the ENSO forcing frequency. Additionally, we see clear secondary peaks associated with the aforementioned combination tones (Fig. 4f). As the slab ocean model integrates both the tropical forcing and stochastic atmospheric variability^5,8^ we see a pronounced reddening of the North Pacific SST anomaly PC1 spectrum (Fig. 4f). This decadal variability is also clearly evident when low-pass filtering the PC1 with a cut-off period of 8 years (red line in Fig. 4e). Given that (i) the simulated PDO-like North Pacific SST anomalies (Fig. 4d) exhibit the characteristic PMM SST anomaly pattern in the Northeastern Pacific (Fig. 3c) and (ii) the high temporal correlation between the observed LF PMM and LF PDO indices (Figs. 1e and 2c) we propose that the PMM and PDO are closely related on decadal timescales (Fig. 5). More general, it is clear that Pacific decadal variability (in form of both PDO and NPGO) shares much commonality with the low-frequency components of both CP ENSO and the PMM (Fig. 2c).

## Conclusions and Discussion

It has been demonstrated in many studies that the PMM is able to induce CP ENSO variability. However, many other possible causes of CP ENSO variability have been proposed as well, such as equatorial air-sea coupled instability, remote forcing from the Atlantic Ocean, and equatorial state-dependent noise in the form of WWEs. Here we show that a close instantaneous relationship exists between CP ENSO and the PMM in the observations both on interannual (high-frequency) and decadal (low-frequency) timescales. To reconcile the co-existence of these different mechanisms, a strong positive feedback needs to be present between CP ENSO and the PMM, in which equatorial CP ENSO SSTs are able to excite a PMM response without any significant delay.

We demonstrate that an idealized CP ENSO SST forcing is able to generate an off-equatorial atmospheric response with a strong projection on the Aleutian Low. This teleconnection acts as an atmospheric bridge to generate an instantaneous PMM response to the tropical forcing via ocean mixed layer dynamics (that is the WES feedback), thereby allowing for a fast Central Pacific-Meridional Mode (CP-MM) positive feedback process (Fig. 5). The CP-MM feedback is the missing puzzle piece that is required to explain the very close instantaneous relationship between these two modes of climate variability on interannual timescales (Fig. 1d). Note that our model is able to simulate meridional mode responses both in the North Pacific and in the South Pacific. Importantly, our results lead to the insight that CP ENSO and PMM cannot be considered two independent dynamical phenomena, which then has important implications for impact attribution studies, such as Murakami *et al*.^38^.

Additionally, the CP ENSO-induced Aleutian Low variability will generate low-frequency North Pacific SST changes, which then is able to explain the close relationship between PMM, CP ENSO, PDO, and NPGO at low-frequencies (Fig. 1e). Therefore, we conclude that our proposed CP-MM feedback mechanism likely operates both on fast and slow timescales. We summarize these pathways and how they fit into our current understanding of the relationships between CP ENSO, the PMM, and Pacific decadal variability in Fig. 5. We expect that including a dynamical ocean in the experimental setup would result in an amplification of the North Pacific low-frequency SST signal.

Importantly, we find that no statistically significant relationship can be established from the observations between EP ENSO events and the PMM (Fig. 2b). The common approach in many studies was to remove the ENSO signal from the PMM via a linear least squares regression approach with either the so-called Cold Tongue Index (SST anomalies averaged from 180°E–90°W and 6°S–6°N) or Niño3.4 (SST anomalies averaged from 170°W–120°W and 5°S–5°N), indices which measure both CP and EP ENSO variability concurrently. We show here that nearly all of the observed co-variability between ENSO and the PMM arises from the CP ENSO signal, therefore utilizing either the Cold Tongue Index or Niño3.4 in a regression approach removes only a small part of the ENSO signal. Hence, we recommend one uses orthogonal ENSO measures to study either ENSO’s relationship with the PMM or ENSO-independent PMM variability. Further, we note that future targeted sensitivity experiments with SST anomaly forcing prescribed at different zonal locations (EP and CP) in a suite of distinct models should help us to disentangle the respective roles of forcing location and model biases in generating characteristic off-equatorial atmospheric circulation anomalies (such as the PMM, Aleutian Low variability, and the NPO) as well as low-frequency SST patterns (such as the PDO and NPGO).

## Methods

We use surface winds from the National Center for Environmental Prediction reanalysis version 1 (NCEP1)^39^ and SSTs from the Hadley Centre Sea Ice and Sea Surface Temperature data set version 1 (HadISST1)^40^. The data was obtained from https://www.esrl.noaa.gov/psd/data/gridded/data.ncep.reanalysis.html and http://www.metoffice.gov.uk/hadobs/hadisst. All anomalies are relative to the 1950–2005 climatology. The Eastern Pacific (EP) and Central Pacific (CP) ENSO indices are defined based on the definition by Ren & Jin^15^, which results in two orthogonal ENSO measures. Note that all the commonly used CP ENSO indices are highly correlated with each other^41^, therefore the specific index choice should not have a qualitative impact on the conclusions presented here. To characterize the PMM we use the raw (for which the ENSO signal is not removed) SST expansion coefficient-based PMM index^21^, which was obtained from http://www.aos.wisc.edu/~dvimont/MModes/RealTime/PMM.RAW.txt. Pacific decadal variability is characterized by utilizing the commonly used PDO^7^ and NPGO^9^ indices, which we obtained from http://research.jisao.washington.edu/pdo/PDO.latest and http://www.o3d.org/npgo/npgo.php. All the data used is for the 1948–2016 period (except for the NPGO index that starts in 1950).

The low-frequency (LF) components of the CP ENSO (LF CP ENSO), PMM (LF PMM), PDO (LF PDO), and NPGO (LF NPGO) indices are obtained via a reconstruction of the lowest-order pair of eigenmodes (eigenmodes 1–2) calculated via Singular Spectrum Analysis (SSA)^42^. For each index these are well separated from the higher-order modes. The high-frequency (HF) components of both the CP ENSO (HF CP ENSO) and PMM (HF PMM) indices are obtained via a reconstruction of the eigenmodes 3–16 of these time series^42^. The eigenmodes beyond are considered noise in this study and discarded due to their short decorrelation timescales on the order of a few months.

We use the Community Earth System Model (CESM 1.2.0)^43^ with the CAM4^44^ atmospheric component (nominally 2° horizontal resolution) partially-coupled (PARCP) to a slab ocean model (SOM). We regress the normalized CP ENSO index^15^ with the SST anomalies to obtain the boundary forcing pattern (Fig. 3a). To get the time evolution of the boundary forcing we multiply an idealized 2.5 years period sinusoidal ENSO forcing (Fig. 3e) with the SST regression pattern and add these anomalies to the SST climatology as we did in previous studies^45–47^. The SST boundary forcing is only prescribed in the equatorial Central Pacific, while the rest of the domain is coupled to the SOM (Fig. 3a). An air-sea coupling strength of 100% means that the AGCM and slab ocean are fully coupled, while a coupling strength of 0% means that the AGCM is forced only by the prescribed SST boundary conditions. Numbers in-between indicate the percentage of the SST boundary forcing that comes from the prescribed data and the SOM respectively. We merge these gradually to avoid any unphysical large spatial gradients in the SST boundary forcing. The PARCP experiment is integrated for 100 years and 5 years cycles are used for the composites (n = 20). Note that the typical EP ENSO mode is not active due to our experimental design: The eastern equatorial Pacific region has only slab ocean dynamics and no coupled Bjerknes feedback.

We calculate the PMM index for the 100 years model experiment output based on a similar method to Chiang & Vimont^21^. The seasonal cycle is removed from the lowest model level winds and SSTs. The anomalous data in the region from 175°E–95°W and 21°S–32°N are weighted by the cosine of the latitude. The PMM in defined as the leading statistical mode of an eigenvalue decomposition of the covariance matrix for the combined (concatenated along the spatial dimension) SST and wind data (commonly referred to by Empirical Orthogonal Function (EOF) analysis^48^). We do not employ any spatial or temporal filtering, however the results are qualitatively the same if we use the filtering methods employed by Chiang & Vimont^21^. The corresponding normalized principal component (PC1) is our model PMM index. To test the robustness of our statistical decomposition we also conduct the above analysis only in the Northern Hemisphere (NH) part of the PMM domain (175°E–95°W and 0°–32°N). The corresponding normalized PC1 is labelled NH PMM index. Note that the PMM index we use for the observations is calculated slightly different by Chiang & Vimont^21^, namely they project the leading spatial pattern obtained from a Singular Value Decomposition (SVD) of the combined SST and wind covariance matrix onto the SST data. However, it is important to note that these two methods capture very similar statistical properties of the data (that is the dominant mode of co-variability between SSTs and surface winds in this spatial domain).

We use the same EOF methodology (but for a single variable) on the composite (n = 20) simulated sea level pressure (SLP) anomalies and on the full (non-composite) SST anomalies separately in the North Pacific (120°E–100°W and 20°N–66°N) to investigate the CP ENSO induced teleconnections. The Multi-Taper-Method (MTM) is utilized to calculate the power spectra of the model PMM, NH PMM, and North Pacific SST PC1^42^.

## Electronic supplementary material

Supplementary Information
