# Supplementary material for: Revisiting the Pacific Meridional Mode
Source: Sci Rep. 2018 Feb 16;8:3216. doi: 10.1038/s41598-018-21537-0 (PMC5816654; doi:10.1038/s41598-018-21537-0)
Supplement: Supplementary file 1 — Supplementary Information [file 41598_2018_21537_MOESM1_ESM.pdf]

Revisiting the Pacific Meridional Mode (Supplementary Information)

Malte F. Stuecker[1,2,*]

[1]Department of Atmospheric Sciences, University of Washington, Seattle, Washington, USA
[2]Cooperative Programs for the Advancement of Earth System Science (CPAESS), University Corporation for Atmospheric Research (UCAR), Boulder, Colorado, USA
[*]stuecker@atmos.washington.edu

ABSTRACT

Contents

1. Figures S1-3

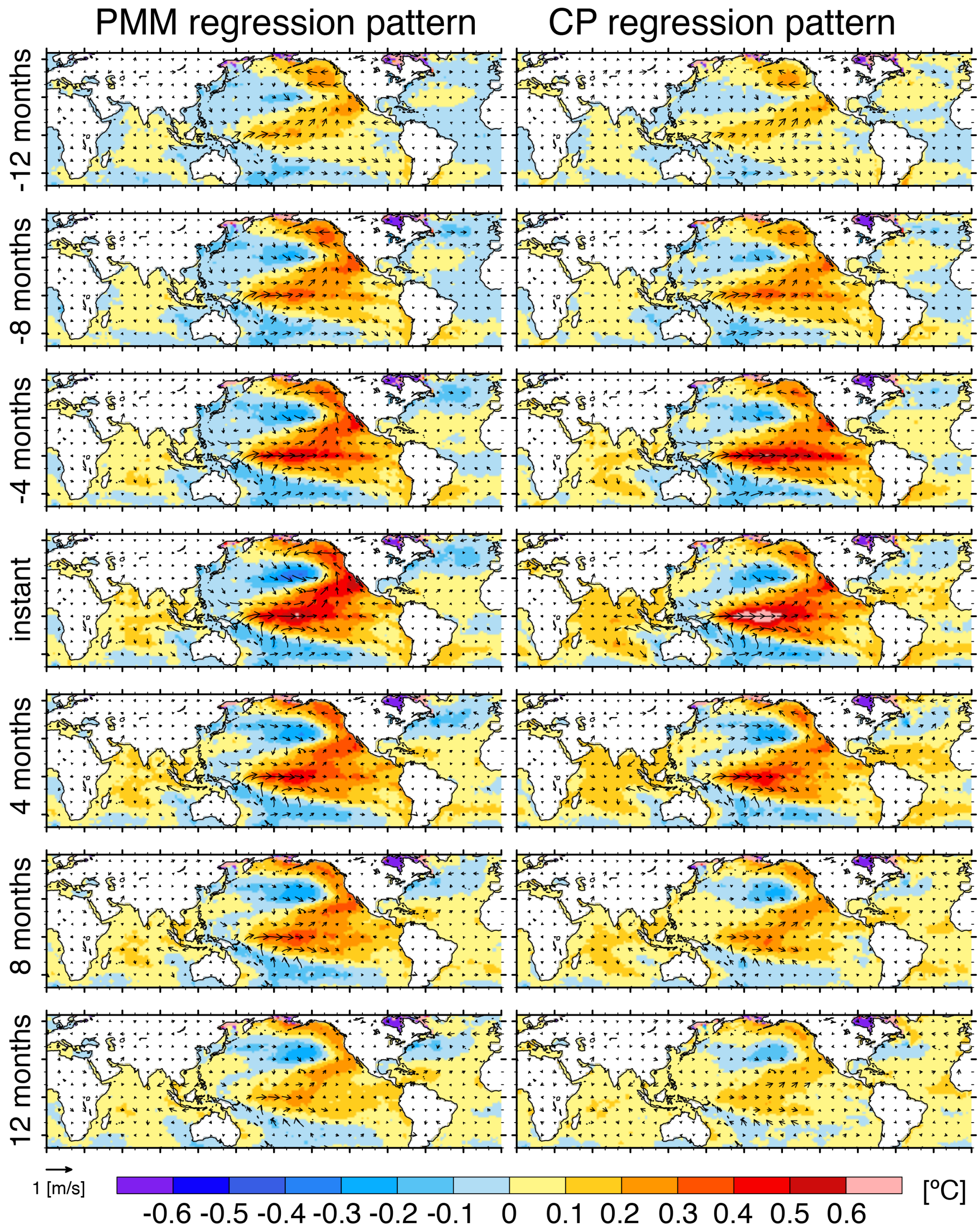

Figure S1. Lead/lag regression patterns (rows for different leads and lags) of observed anomalous surface winds and SST with the normalized PMM (left column) and CP ENSO (right column) indices.This figure was created using NCAR Command Language Version 6.4.0 (http://dx.doi.org/10.5065/D6WD3XH5).

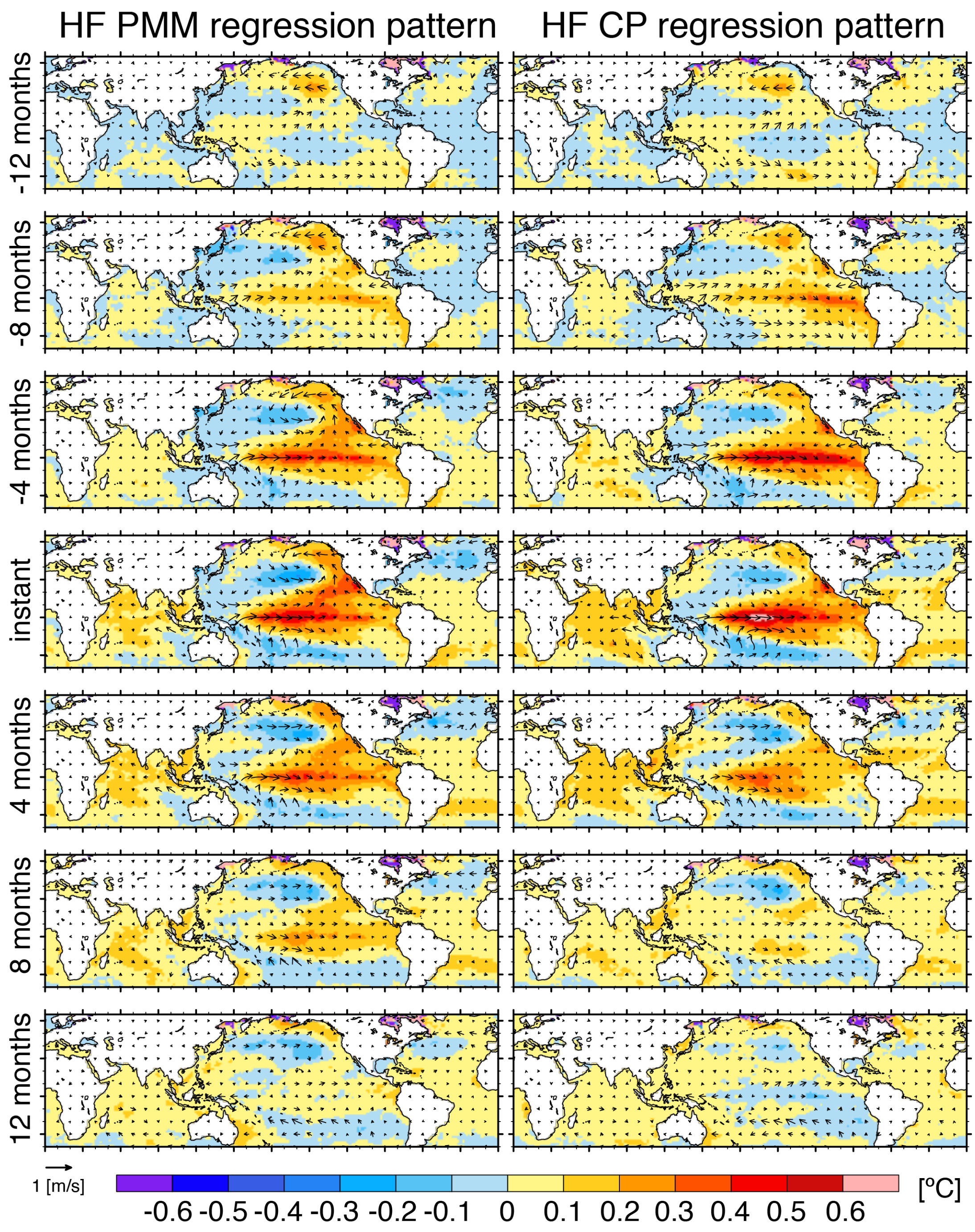

Figure S2. As Fig. S1 but for the high frequency (HF) components of both indices. This figure was created using NCAR Command Language Version 6.4.0 (`http://dx.doi.org/10.5065/D6WD3XH5`).

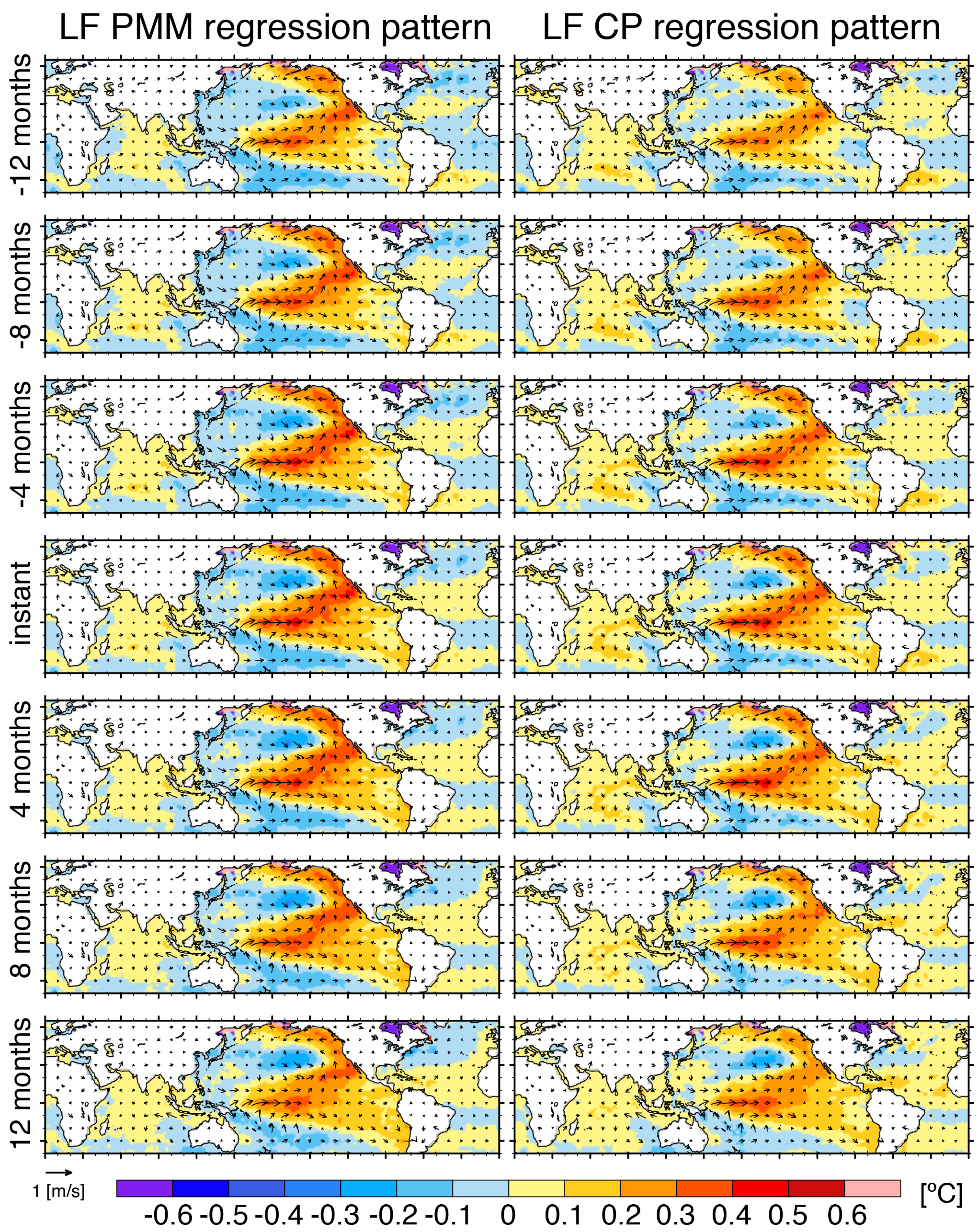

Figure S3. As Fig. S1 but for the low frequency (LF) components of both indices. This figure was created using NCAR Command Language Version 6.4.0 (http://dx.doi.org/10.5065/D6WD3XH5).
